# Ultrasound triggered image-guided drug delivery to inhibit vascular reconstruction via paclitaxel-loaded microbubbles

**DOI:** 10.1038/srep21683

**Published:** 2016-02-22

**Authors:** Xu Zhu, Jun Guo, Cancan He, Huaxiao Geng, Gengsheng Yu, Jinqing Li, Hairong Zheng, Xiaojuan Ji, Fei Yan

**Affiliations:** 1 Department of Cardiology, Children’s Hospital of Chongqing Medical University, Chongqing, China; 2 Ministry of Education Key Laboratory of Child Development and Disorders, Chongqing Key Laboratory of Pediatrics, Chongqing, China; 3 Department of Pediatrics, Mianyang Central Hospital, Mianyang, Sichuan, China; 4 Department of Radiology, 324 Hospital of the PLA, Chongqing, China; 5 Paul C. Lauterbur Research Center for Biomedical Imaging, Institute of biomedical and Health Engineering, Shenzhen Institutes of Advanced Technology, Chinese Academy of Sciences, Shenzhen, China; 6 Shenzhen Key Laboratory of Nanobiomechanics, Shenzhen Institutes of Advanced Technology, Chinese Academy of Sciences, Shenzhen, China

## Abstract

Paclitaxel (PTX) has been recognized as a promising drug for intervention of vascular reconstructions. However, it is still difficult to achieve local drug delivery in a spatio-temporally controllable manner under real-time image guidance. Here, we introduce an ultrasound (US) triggered image-guided drug delivery approach to inhibit vascular reconstruction via paclitaxel (PTX)-loaded microbubbles (PLM) in a rabbit iliac balloon injury model. PLM was prepared through encapsulating PTX in the shell of lipid microbubbles via film hydration and mechanical vibration technique. Our results showed PLM could effectively deliver PTX when exposed to US irradiation and result in significantly lower viability of vascular smooth muscle cells. Ultrasonographic examinations revealed the US signals from PLM in the iliac artery were greatly increased after intravenous administration of PLM, making it possible to identify the restenosis regions of iliac artery. The *in vivo* anti-restenosis experiments with PLM and US greatly inhibited neointimal hyperplasia at the injured site, showing an increased lumen area and reduced the ratio of intima area and the media area (I/M ratio). No obvious functional damages to liver and kidney were observed for those animals. Our study provided a promising approach to realize US triggered image-guided PTX delivery for therapeutic applications against iliac restenosis.

Congenital or acquired cardiovascular morbidity stenosis brings serious threats to human health. Clinically, percutaneous transluminal angioplasty and stenting were widely used for treatments of vascular stenosis. However, restenosis often occurs due to the vascular structure contusion during operation^1^,^2^. The main pathophysiological mechanisms involved in restenosis mainly attribute to endothelial injury and neointimial hyperplasia^3^. Following endothelial injury, a layer of platelets and fibrin are deposited over the damaged endothelium. Within hours to days, inflammatory cells begin to infiltrate the injured area. After that, vascular smooth muscle cells (VSMCs) located in the vessel media commences DNA synthesis. These activated VSMCs migrate through the internal elastic lamina towards the luminal surface. The continued replication and the production of extracellular matrix by these cells result in a neointima and an increase in the intimal thickness^3^.

To date, various pharmacological agents were developed for the prevention and treatment of restenosis^4^,^5^. Paclitaxel (PTX), an amitotic inhibitor widely used in cancer chemotherapy, has been recognized as a promising drug for intervention of vascular reconstructions in recent years^6^,^7^,^8^,^9^,^10^. Its several chemical properties, such as hydrophobicity, ability to concentrate into the arterial intima layer and prolonged effect on cells even after brief exposure periods, make it an ideal drug for intervention of vascular reconstructions. Still, the shortage that is significant toxic effects on liver and kidney functions are still unavoidable^11^. In order to address this issue, local delivery of PTX through injection catheters, balloon catheters and coated balloons has developed^12^,^13^. Drug-eluting techniques (DES), such as balloons and stents covered with PTX, have also shown promising progress in decreasing restenosis and revascularization rates^14^,^15^. However, studies have revealed a possible increase in the rate of stent thrombosis with DES due to decreased endothelialization of stented regions^16^,^17^. In addition, the polymer coating on DESs may induce inflammatory responses that could also induce stent thrombosis^18^. These limitations have prompted us to search for other local drug delivery modalities to inhibit restenosis and to avoid systemic adverse effects.

Microbubbles (MBs) are small air- or gas-filled microspheres with specific acoustic properties that make them be useful as US contrast agents for sonographic examinations. Besides, MBs are also important drug delivery vehicles that can stably carry drugs in the blood circulation^19^. Under US irradiation, drugs are able to be controllably released from MBs and to be delivered into the targeted regions. In this study, we prepared the PTX-loaded MBs (PLM) and investigated the efficacy of PLM combined with US against restenosis in rabbit iliac balloon injury model. The functional damages to liver and kidney of these animals were also demonstrated.

## Materials and Methods

### Preparation of PLM

Lecithin (Sinopharm Chemical Reagent Ltd. Co., Shanghai, China), cholesterol (KeLong Chemical Reagent Ltd. Co., Chengdu, China) and PTX (Chengdu Yuancheng Biotechnology Ltd. Co., Chengdu, China), all in ethyl ether (3 : 1 : 0.3, weight ratio), were added to the reaction bottle. The mixture was kept in water bath at 55 °C by a rotary evaporator until the ethyl ether was completely evaporated. Then a given amount of PBS, PEG 4000, dextran 40000 and span 80 was added and mixed. After that, the air was displaced with the nitrogen gas. After being vibrated and centrifuged at 300 rpm for three minutes, 125 μl of 3% human serum albumin was added to the resulting PLM. For the control lipid MBs (LM), the same procedure was performed instead of PTX addition.

### Characteristics of PLM

The size and size distribution of the control MBs and PLM were determined by an optical particle counter with a 0.5 μm diameter lower detection limit (Accusizer 780; Particle Sizing Systems, Santa Barbara, CA, USA). In brief, 100 μl of MBs were detected and the experiments were repeated 3 times. In order to determine the drug encapsulation efficiency (EE), a given amount of PLM was destructed by adding 1% Triton X-100 and extracted by 1 ml chloroform. The extraction was dried and dissolved with 1 ml methanol. 20 μl of samples were used for analysis with an Agilent 1200 HPLC system (Agilent Technologies, USA) equipped with an Eclipse XDB-C18 (4.6 × 150 mm, 5 μm particle size). The methanol and water mixture (70 : 30 in volume) was used as the mobile phase. The flow rate was set at 1.0 ml/min and the detection wavelength was at 227 nm. As for the stability test, the MB concentrations, size and drug EE were examined after the freshly prepared PLMs were left to stand for 0.5, 6, 18 or 32 hours.

### Drug release efficiency under US irradiation

1 ml of PLM was placed in the 1.5 ml eppendorf tube sealed with parafilm (the lid was removed). The tube was inverted and positioned onto the surface of the transducer. The gap between the tube and transducer was filled with the ultrasonic coupling agent. The following US parameters were used: 1 MHz transducer, 1.5 MPa US intensity. The irradiation time is 10 s. After US treatment, these tubes were centrifuged for 20 min at 15,000 g. The supernatant was transferred into a new tube and extracted by chloroform. The PTX amounts were determined by HPLC as stated above. The released efficiency was calculated according to the following formula: The drug release efficiency (%) = free PTX/total PTX × 100.

### Cell culture and viability detection

Rat aortic VSMCs were isolated by enzymatic dispersion according to the previous report with some slight modification^20^. Cells were cultured in Dulbecco’s Modified Eagle’s media/F-12 supplemented with 10% fetal bovine serum (FBS), 100 U/ml penicillin and 100 mg/ml streptomycin at 37 °C in an incubator with 5% CO_2_. After a 3- to 5-generation subculture, the VSMC cells were seeded on 96-well plates with 5000 cells/well and allowed to grow overnight. The cells were divided into four groups according to the different treatment as follows: 1) PBS, no US and MBs were used; 2) only US treatment; 3) LM + US; 4) PLM (containing 10 μg PTX) without US; and 5) PLM + US. 1.0 MHz US transducer was used. The US parameter was set at 1.5 MPa US intensity for 10 s. After treatment, these cells were further cultivated for 24 h, followed by detection with CCK-8 reagent according to the production instruction.

### Animal model and *in vivo* US imaging

New Zealand White rabbits, at the age of two months with about 0.7 kg body weight, were obtained from the Animal Laboratory Center at Chongqing Medical University. The animals were fed water and chow ad libitum throughout the experiment. All animal experimental protocols were reviewed and approved by the Chongqing Medical University Animal Care Committee. The methods were carried out in accordance with the approved guidelines. Iliac restenosis were modeled according to the previous report^21^. Briefly, after the rabbits were anesthetized with 10% chloraldurate (3 ml/kg), a 2 F Fogarty embolectomy catheter (Baxter Edwards Healthcare Corp., Irvine, CA) was advanced from the femoral artery to the iliac artery. The saline-inflated balloon was moved backward and forward within the iliac arterial lumen for 3 times to produce a substantial endothelial injury. Then 80,000 U of penicillin were administrated by muscle injection for three days to prevent infection. Three weeks post the iliac balloon injury, 8 × 10^7^ PLM or Sonovue were injected into the auricular veins of rabbits. The US imaging transducer was positioned the iliac arterial regions which were received balloon injury. Ultrasonography was performed using an ESAOTE Imaging System with the following parameters: probe, LA522; grain, 55% dB; focal depth, 10 mm; transmit power, 17%; mechanical index, 0.11; dynamic range, 12 dB, frame rate, 10 Hz; and center frequency, 3–9 MHz.

### *In vivo* local drug delivery mediated by PLM and US

Twelve New Zealand White rabbits were used and modeled as stated above. After three weeks, these animals were randomly divided into four groups: 1) no treatment; 2) PLM; 3) PTX + US; 4) PTX + LM + US and 5) PLM + US. PLM or free PTX was injected via ear margin veins and the dose of PTX was kept constant with 125 μg for each rabbit. The MB perfusion was monitored by an US imaging probe as described above and the local MB destruction at the balloon injured iliac arteries was performed with a 1 MHz low frequency focal transducer. The US power was set at 1.5 MPa US intensity and the US time was set for 10 seconds. 2 h after US treatment, the artery fragments received US irradiation were isolated and homogenated, followed by centrifugation for 15 min at 15,000 g. The supernatant was removed and the PTX concentrations were detected by ultraviolet-visible spectrometry.

### *In vivo* inhibition effect of PLM on iliac restenosis

Fifty-five New Zealand White rabbits were used to evaluate the *in vivo* inhibition effect of PLM on iliac restenosis. Three weeks post the iliac balloon injury, the rabbits were randomly divided into five groups: 1) PLM + US; PLM (containing 125 μg PTX) was administrated by intravenous injection via ear margin veins. 2) LM + US; the equal concentration of plain MBs were used. 3) PTX + US; the equal amount of PTX as PLM was injected. 4) only US; 5) control; no US and MBs were used. The balloon injured iliac arteries were received US irradiation as stated above.

### Histological analysis

The animals were sacrificed one week after US treatment and the balloon injured iliac arteries were harvested. The tissue samples were fixed in 4% paraformaldehyde for 24 h and embedded in paraffin. The sections (5 μm thick) were cut with a paraffin section machine (RM2235; Leica, Heidelberg, Germany), followed by staining with haematoxylin/eosin staining (Rowley Biochemical Institute, Danvers, MA, USA). Images of the cross-sections of the vessels were photographed with an Olympus BX-51 optical microscope. After the digitized images were obtained, areas were measured and calculated by the Meta-Morph image analysis system for each cross-section. The area of lumen, the area of intima area and the tunica media were determined. The ratio of the intima area to the media area (I/M ratio) was calculated for assessing the degree of intimal hyperplasia.

### Immunohistochemical analysis

The expression of α-smooth muscle actin (α-SMA) was used to identify the proliferated smooth muscle cells in the neointima. In brief, sections prepared from paraffin-embedded samples were immunohistologically stained with α-SM actin immunohistochemical kit (Beijing Biosynthesis Biological Co., Ltd). The immunohistochemical score (IHS) was performed to evaluate the expression level of α-SMA according to the previous report^22^. In brief, IHS was calculated by multiplying an approximation of the percentage of positively stained cells by an estimate of the staining intensity. The approximation of percentage of positive cells between 0–25% of positively stained cells was scored as 1, 26–50% as 2, 51–75% as 3, and 75–100% as 4. The staining intensity was set as follows: 0 = no expression, 1 = weak, 2 = moderate, and 3 = strong. The number of positive cells per section was counted in five random fields (×400 magnification).

### Examinations of liver and kidney functions

30 Young New Zealand rabbits were randomly divided into three groups, including normal group, sham group and US irradiation group. The iliac artery stenosis model was established. As for the animals of US irradiation group, the PLM was administrated and received US irradiation as stated above. One week later, the blood samples (1 ml for each rabbit) were collected through ear marginal veins. The total protein (TP), aspartate aminotransferase (ALT), aspartate aminotransferase (AST), glutamic pyruvic transaminase (GPT), alkaline phosphatase (ALP), L-lactate dehydrogenase, blood urea nitrogen (BUN), creatinine (CREA) and uric acid (URCA) were determined with HITACHI 7600(JAPAN) automatic biochemistry instrument.

### Statistical analysis

Data are expressed as mean ± standard deviation. One-way analysis of variance (ANOVA) was used to compare the variables among different treatment groups *in vitro* experiment. The two-way ANOVA was performed to determine the differences between treated animals and control animals. Analysis was performed with SPSS 18.0 software. P < 0.05 was considered as the level of significance.

## Results

### Preparation and characterization of PLM

**Figure 1:**
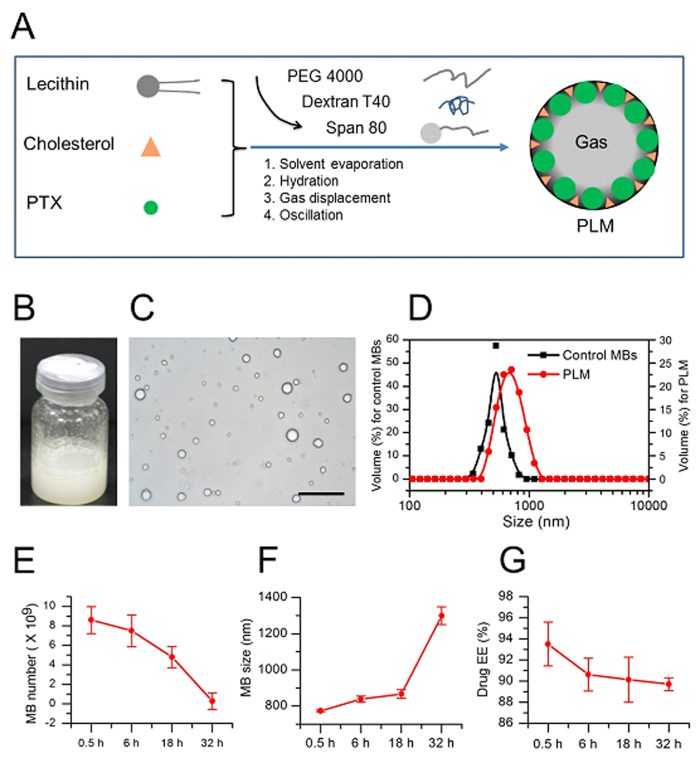
Characterization of PLM. (**A**) Schematic diagram of a PLM constructed for drug delivery. PTX was encapsulated into the shell of gas-filled MBs. (**B**) A typical bottle of PLM after mechanical activation. (**C**) An optical microscope of PLM, scale bar = 10 μm. (**D**) The bubble size distributions of the control MBs and PLM. (**E–G**) The MB concentrations, bubble size and drug EE of PLM after the freshly prepared PLMs were left to stand for 0.5, 6, 18 or 32 h.

PLM was prepared by thin-film rehydration and mechanical vibration method through the following several steps, film-forming, solvent evaporation, hydration, gas displacement and mechanical vibration (Fig. 1A). The resulting PLM appeared milky and had a uniform distribution (Fig. 1B,C). The PLM was submicron size with an average particle diameter of 772.9 ± 6.2 nm, significantly larger than of the plain MBs (456.5 ± 5.8 nm) (Fig. 1D). Typically, it produced (8.64 ± 1.38) × 10^9^ bubbles per milliliter and achieved 93.51 ± 2.07% PTX encapsulation efficiency. The stability test including PLM concentrations, bubble size and drug leakage revealed there were gradually decreased bubble concentrations and increased bubble size when PLM were left to stand for a period of time (0.5 h to 32 h) (Fig. 1E,F). Also, a few drug leakage (<5%) was found after 32 h (Fig. 1G). Nevertheless, PLM was relatively stable since there was still over 90% drug EE after 32 h.

### Drug release efficiency and cell proliferation inhibition

**Figure 2:**
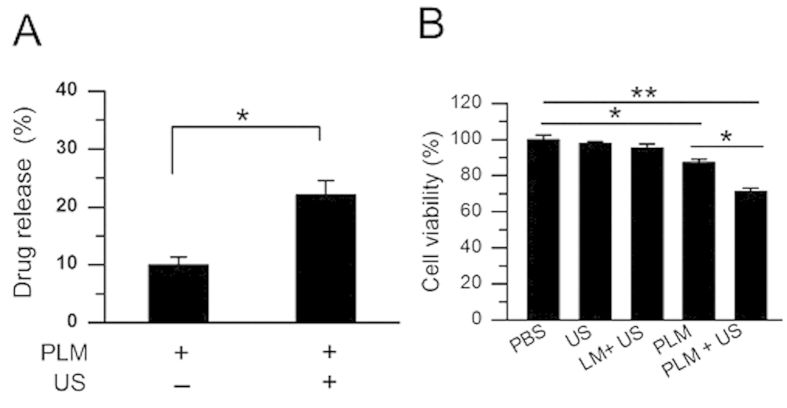
Drug release and *in vitro* cell growth inhibition. (**A**) US promoted PTX release from PLM *in vitro*. *P < 0.05. (**B**) Cell viability of VSMC after treatment with PBS, US, LM + US, PLM or PLM + US. After different interventions, the cells were cultured for another 24 h at 37 °C. Cell viability was determined by CCK-8 assay. *P < 0.05,**P < 0.01.

The drug release efficiencies of PLM were determined with US or without US exposure. As shown in Fig. 2A, there was just 9.94 ± 1.41% of PTX to be released when no US was used. By contrary, significantly higher drug release efficiency could be obtained, achieving 22.05 ± 2.44% under US irradiation. Since vascular smooth muscle cell (VSMC) proliferation causes intimal thickening in restenosis, we first examined the cell proliferation inhibition effects of PLM combined with US on VSMCs. Figure 2B demonstrated that the cell viability was 97.71 ± 1.15% and 95.28 ± 2.34% for only US treatment and LM + US, respectively. In contrast, treatment using of PLM without US had an 87.16 ± 1.93% cell viability, lower than that of PBS control treatment (P < 0.05). As expected, the combination of PLM and US showed the highest cell inhibition effect, achieving 67.93 ± 2.52% cell viability (p < 0.05 vs PLM and p < 0.01 vs PBS).

### *In vivo* US imaging of PLM

**Figure 3:**
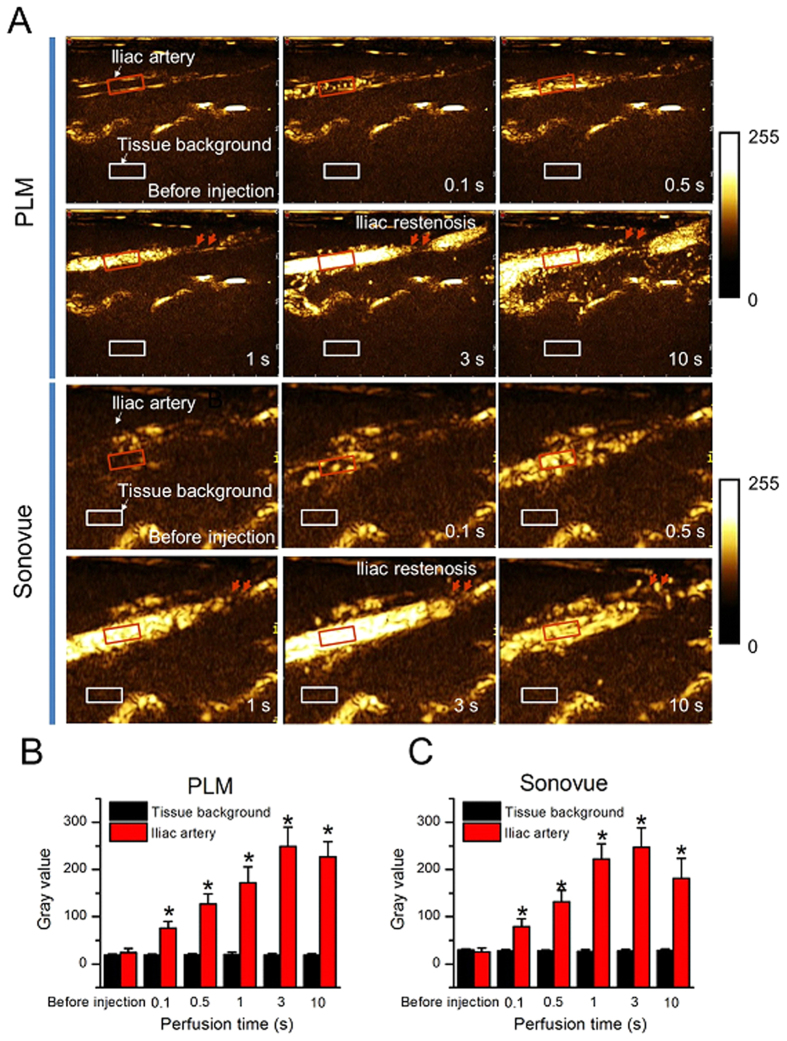
US imaging of PLM and Sonovue in the rabbit iliac arteries. (**A**) The rabbits were administrated with PLM (Top two rows) or Sonovue (bottom two rows) at the same bubble concentrations. After bubble perfusion, US images were obtained at 0.1 s, 0.5 s, 1 s, 3 s or 10 s. (**B**,**C)** The quantitative analysis of US signal intensities of selected regions after PLM or Sonovue perfusion at different times. The arrows are for the regions of iliac artery restenosis. Red boxes are for selected regions of the rabbit iliac arteries. White boxes for selected regions of tissue background. *P < 0.01.

Next, we further examined the *in vivo* US imaging capability of PLM in the rabbit iliac restenosis model. Figure 3A showed that PLM had a comparable US contrast capability with the commercial Sonovue agent. Both of them exhibited a fast iliac artery perfusion within a couple of seconds. There had no US signals in the iliac artery before injection of MBs. After administration of PLM or Sonovue, the US signals greatly increased in the iliac artery. The gray values from PLM scattering were 24.05 ± 8.54, 75.91 ± 14.43, 126.74 ± 21.42, 171.56 ± 34.21, 249.04 ± 40.71 and 226.78 ± 32.42 after PLM perfusion for 0.1 s, 0.5 s, 1 s, 3 s and 10 s, respectively (Fig. 3B). Similarly, the gray values from Sonovue scattering were 25.37 ± 8.27, 78.68 ± 16.75, 131.37 ± 24.38, 222.32 ± 32.58, 247.02 ± 45.35 and 181.20 ± 43.65 at the corresponding perfusion time, respectively (Fig. 3C). In contrast, the gray values from tissue background were not significantly changed after injection of PLM or Sonovue. Notably, the regions of iliac restenosis artery could be observed about 1 s after MB perfusion, indicating the feasibility of US imaging guided drug delivery.

### Drug local delivery mediated by PLM and US

**Figure 4:**
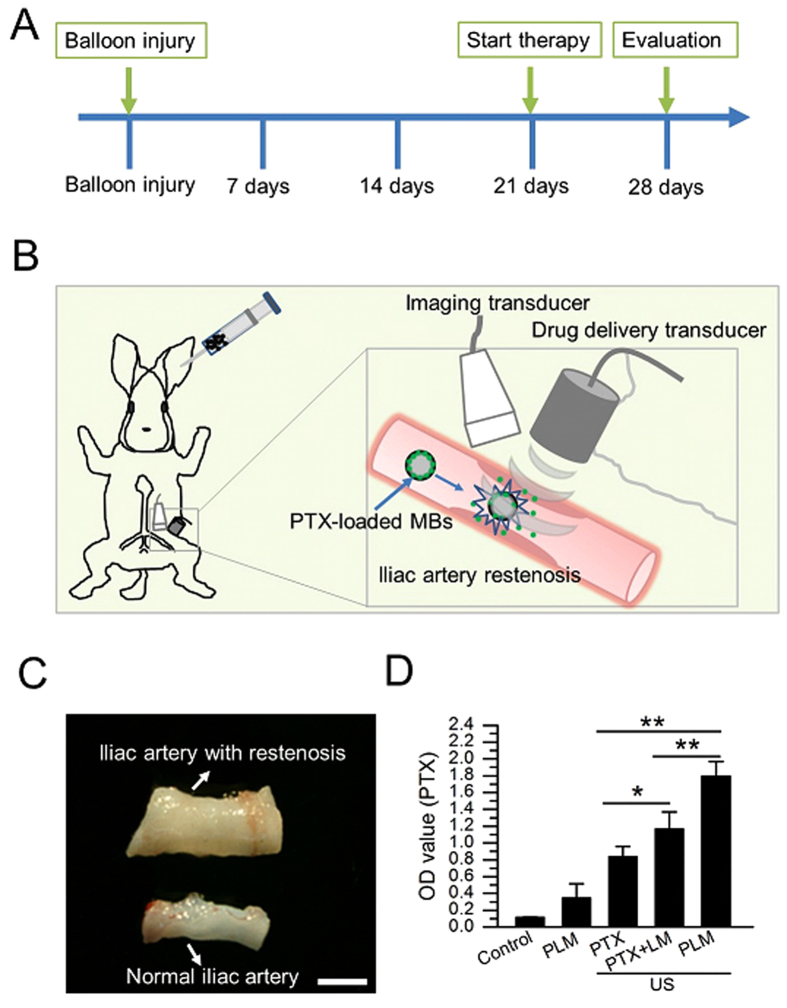
Overview of the experimental protocol. (**A**) Experimental protocol over time (days) is shown, with each vertical line on the x-axis representing a time point at intervals of one week. The arrows represent the time point of animal treatments, corresponding to balloon injury, start therapy and evaluation, respectively. (**B**) Diagram shows the experimental setting, with the rabbit lying supine and the site of iliac artery restenosis received with US imaging and therapy. Partial enlarged drawing of iliac artery restenosis which was perfused with PLM and received with therapy under US imaging is shown (inset). (**C**) The isolated fragments of the iliac artery with restenosis and without restenosis were presented. Scale bar: 2 mm. (**D**) The *in vivo* drug local delivery efficiency was determined by examining PTX amounts in the isolated iliac arteries.

To further determine whether PLM and US can promote local drug delivery, an experimental scheme over time was shown in Fig. 4A. The iliac restenosis animals were developed through balloon injury method, followed by treatment with PLM plus US after three weeks in these animals. To realize imaging-guided drug local delivery, the experimental setting was setup as Fig. 4B, where the rabbit was lying supine and the iliac restenosis region was positioned with two US transducers. The one is high frequency imaging transducer and the other is low frequency drug delivery transducer. Upon the PLM was injected into the ear margin veins, the imaging transducer would be used to observe and identify the restenosis region of iliac artery. It could favor to orientate US focus accurately for the drug delivery transducer by examining whether the US signal intensity from PLM scattering does decrease or not. After receiving US irradiation, the iliac restenosis artery and the control artery fragments were isolated and homogenated (Fig. 4C). The amounts of PTX in the homogenate of iliac restenosis artery were determined. Obviously, Fig. 4D showed there were 5.20-, 2.13-, 1.54-fold increase of PTX amounts in the artery treated with PLM + US than that of artery treated with PLM without US, free PTX+US or free PTX+LM+US groups (P < 0.01).

### *In vivo* inhibition effect of PLM on iliac restenosis

**Figure 5:**
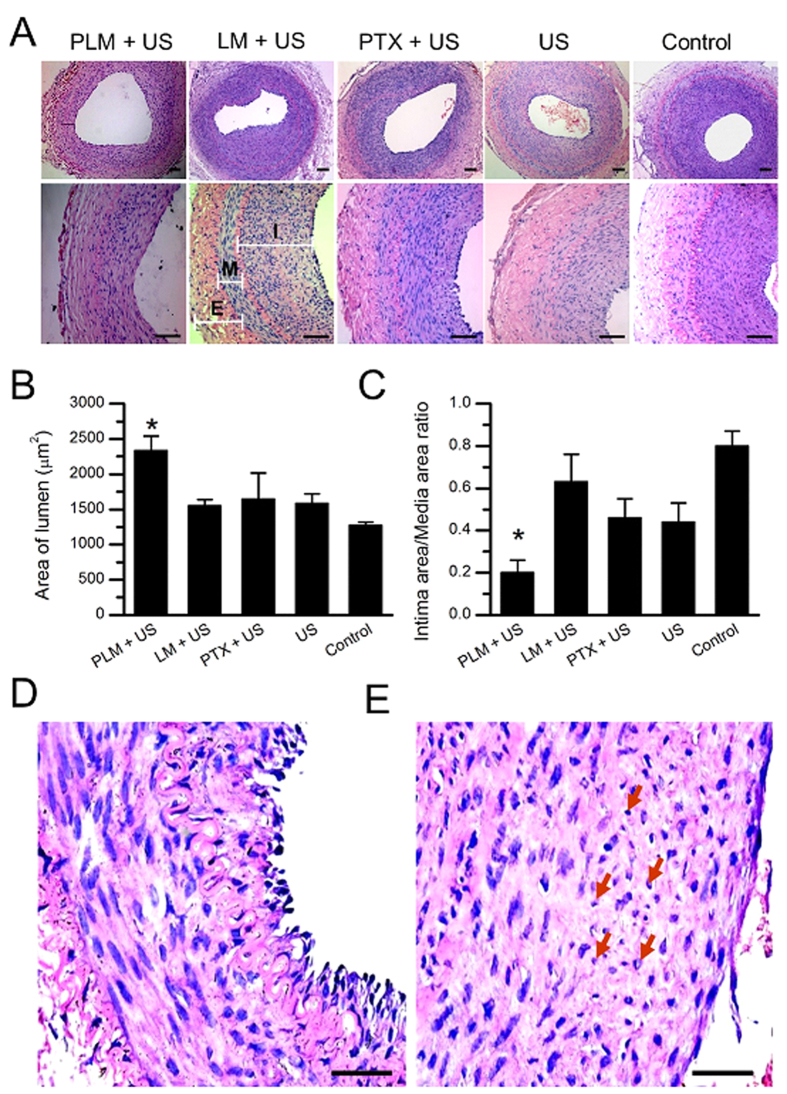
Efficacy assessment of iliac artery restenosis. (**A**) Representative H&E-stained arterial cross-sections from different treatment groups in a rabbit model of iliac artery restenosis (top). Zoomed-in H&E-stained arterial cross-sections highlight the different vascular remodeling from the different treatment groups (bottom). I, intima; M, media; E, externa. Scale bar, 100 μm. (**B,C**) Quantitative analysis of luminal area and intima-to-media area ratio from the different treatment groups. (**D,E**) Representative zoomed-in H&E-stained arterial cross-sections from PLM + US group and control group highlight the significant reduction of disorder cell arrangement and irregular cell shape (red arrows). Scale bar: 100 μm.

To determine the inhibition effect of PLM against restenosis *in vivo*, the restenosis inhibition effects were evaluated one week after intervention treatment. Figure 5A showed the representative histological sections of the artery at the injured site after treatment. Neointimal hyperplasia at the injured site was significantly inhibited by PLM and US (left panel) compared with the control (right panel). The PTX and US showed no significant effect on neointimal formation, similar with the effects of LM+US and only US-treated groups. Also, there was a significantly larger lumen area to be observed in the animals received PLM + US treatment. Quantitatively, there was 2337.03 ± 206.05 μm^2^ of lumen area for the animals received with PLM+US, significantly higher than that of 1274.35 ± 43.78 of lumen area for the control animals. No significant increase of lumen area was found in these animals received with PTX+US, LM+US and only US treatment, with 1547.25 ± 90.93, 1642.83 ± 374.18, 1587.97 ± 132.16, respectively (Fig. 5B). Figure 5C demonstrated the intimal area/media area (I/M) ratios on the day 28 (a week after intervention treatment) among the groups. PLM+US treatment resulted in the most significant reduction in I/M ratio compared to the control and other treatment groups. Notably, the significant reduction of disorder cell arrangement and irregular cell shape were observed in the PLM and US treatment group (Fig. 5D) in comparison with that of the control group (Fig. 5E).

### Immunohistochemical analysis

**Figure 6:**
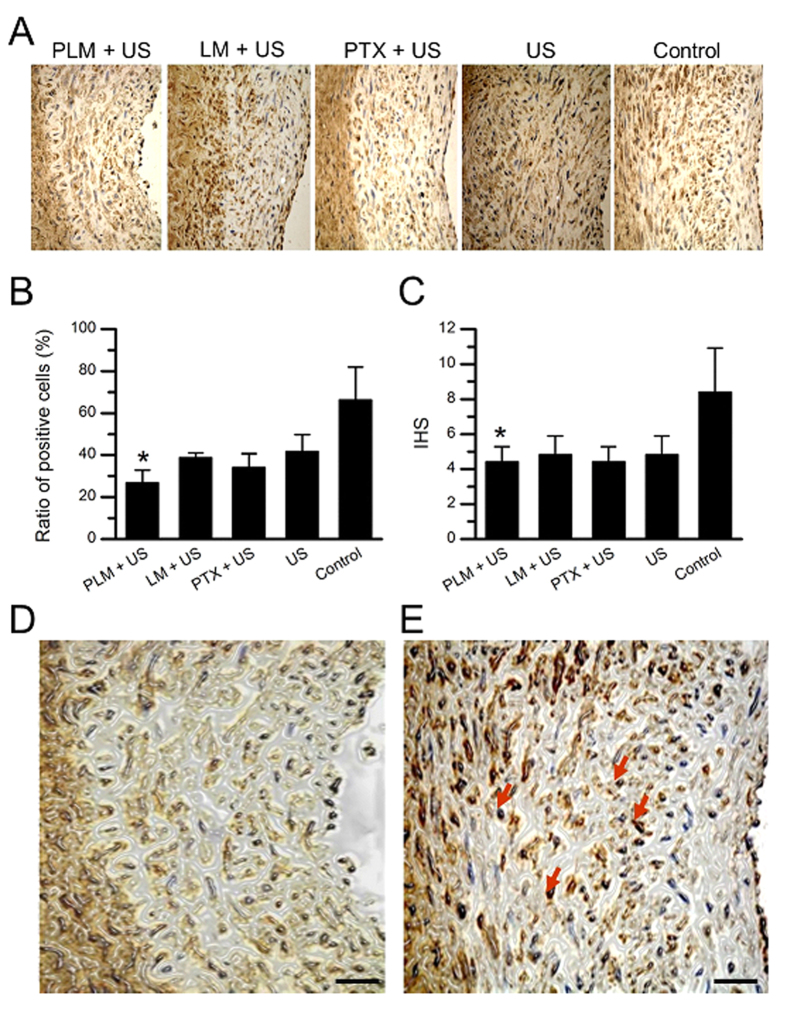
Immunohistochemical staining for α-SMA proteins. (**A**) Representative anti-α-SMA antibody-stained arterial cross-sections from different treatment groups in a rabbit model of iliac artery restenosis. Scale bar, 50 μm. (**B,C**) Quantitative analysis of α-SMA-positive cells and the immunohistochemical score (HIS) from the different treatment groups. Representative zoomed-in H&E-stained arterial cross-sections from PLM + US group (**D**) and control group (**E**) highlight the significant reduction of intimal hyperplasia and irregularly shaped cells (red arrows). Scale bar: 100 μm.

Since α-SMA was used to identify the proliferated smooth muscle cells in the neointima, we further examined the expression of α-SMA through immunohistochemical staining. Figure 6A showed the representative immunohistological sections of the artery at the injured site at 7 days after treatment. The α-SMA positive cells appear brownish black in the cytoplasm which could be seen in both the tunica intimal and the tunica media. Most of the cells in the control artery were the positive ones (left panel). By contrary, the number of positive cells from PLM+US treated artery was significantly decreased in comparison with the control and other intervention groups (P < 0.05) (Fig. 6B). The HIS score was 4.40 ± 0.89 in PLM+US group, which had significantly lower than that of the control artery with a 8.40 ± 2.51 HIS score (P < 0.05) (Fig. 6C). The magnified sections from PLM + US treated artery and control artery were presented in the Fig. 6D,E, showing significantly stronger staining in the control artery in comparison with PLM + US treated artery.

### Safety of PLM+US treatment

In order to assess the safety of PLM+US treatment, we examined the liver and kidney functions on these animals. Table 1 showed the amounts of the total protein (TP), aspartate aminotransferase (ALT), aspartate aminotransferase (AST), alkaline phosphatase (ALP), glutamic pyruvic transaminase (GPT) and L-lactate dehydrogenase (LDH) did not significantly changed in the PLM+US-treated animals, compared with normal and non-intervention control rabbits. It indicated treatment with PLM+US had not functional liver injury on the intervention animals. Similarly, no significant changes were found in the amounts of blood urea nitrogen (BUN), creatinine (CREA) and uric acid (URCA) for these animals (Table 2), demonstrating relatively normal kidney function after PLM+US treatment.

## Discussion

It is widely believed that the mechanisms of vascular restenosis mainly attribute to VSMCs proliferation and neointimal hyperplasia after the endothelial injury^23^,^24^. Documents have demonstrated PTX can inhibit VSMCs proliferation and reduce the neointimal hyperplasia^6^,^7^,^8^,^9^,^10^. In our study, we used MBs, an ultrasound contrast agent, to act as drug carriers to prepare the PLM and successfully achieved US-visible PTX delivery for treatment of vascular restenosis in the rabbit iliac restenosis model. The US-visible drug delivery platform with PLM possesses many advantages. Firstly, the poor water solubility of PTX can be overcome by encapsulating them into the lipid shells of MBs^25^. Secondly, MBs, as a contrast agent only stayed in the blood pool, can limit the drugs in the circulation due to their large particle size. This would greatly increase the bioavailability of PTX and decrease the potential damage to other unwanted tissues or organs. More importantly, taking advantages of MBs in both US imaging and US-triggered drug release, it is possible to achieve an US-visible drug delivery to the local vascular restenosis site.

Although US-triggered drug delivery using MBs holds a great promise for many disease treatments due to its minimal invasiveness, locally enhanced drug delivery and proven safety record. The limited drug loading capacity of lipid monolayer MB shells has become a major obstacle to their therapeutic utility^26^. In our current study, we optimized the formulation and preparation process which achieved 93.51 ± 2.07% of PTX encapsulation efficiency. The high PTX encapsulation efficiency might not only contribute to the optimized formulation, but also contribute to the preparation process that produced relative small bubble size than that of the conventional MBs. In addition, some functional materials including PEG4000, Dextran T40 and Span 80 were used when preparing PLM. PEG4000 is a polymer, it can form a water barrier on the surface of the phospholipid membrane, and prolong the retention time in the blood circulation, and increase the stability of PLM. Dextran T40, a high permeability sugar, can increase the viscosity of the solution, and the hydrogen bond on the sugar can also interact with the hydrogen bond of the surface of PLM, making PLM be more stable. Span 80, as a foaming agent, can stabilize lipid membranes and increase the yield of PLM. These functional materials further improve the stability of PLM and partly contribute to high PTX encapsulation efficiency.

The results of the drug release experiments showed that there had a significant higher concentration of PTX released into the solution after US irradiation (Fig. 2A), indicating PLM had a good US-triggered drug release capability. Meanwhile, the VSMC proliferation experiments further demonstrated a significant higher cell proliferation inhibition effect when these cells were received PLM + US treatment (Fig. 2B). The enhanced cell proliferation inhibition effect may partly attribute to the increased drug concentrations due to the US-mediated PLM destruction. It should also be pointed out that the accompanying physical mechanical energy as the microstreaming, micro-jet and shave wave from MB cavitation would further favor these US-released drugs to enter into the targeted cells. Numerous evidences have showed that the sonaporation effects would produce many nanoscale or microscale holes in the cell membrane surface when cells are exposed to US irradiation in the presence of MBs. Thus results in greatly increased cell membrane permeability^27^,^28^. Therefore, the simultaneous contributions from the locally released drugs and increased permeability can account for the enhanced PTX efficacy against VSMC proliferation.

Data from ultrasonography suggested that PLM had an excellent US imaging capability in the iliac artery. It would not only facilitate to identify the region where restenosis occurs, but also would make it possible to track PTX delivery *in vivo* in a real-time manner. Combined with low frequency focal US irradiation, a spatio-temporally controllable drug delivery could be achieved. Although it is inevitable that a part of PTX would be released at a distal place other than the stenosis place due to the high blood flow speed in artery, there were more PTX released at the stenosis place since the PLM were busted at the stenosis place (Fig. 4D). Also, the cavitation effect would produce sonoporation in the artery wall, favoring the released PTX to penetrate into the artery wall. To date, drug-eluting stents are considered the best way to treat restenosis currently, but they may reduce the flexibility of the artery and limit the repeatability of the procedure^16^,^29^. Other various methods, such as double-balloon catheters and porous balloons, are also widely investigated for carrying drugs on the surface of the balloon to inhibit restenosis. However, all of them are still difficult to achieve the controllable drug release, especially under the real-time imaging-guided conditions. Our study provided a promising approach to realize US image-guided PTX delivery for therapeutic applications for iliac restenosis.

The results of *in vivo* animal experiments indicated that the lumen area of the iliac artery was obviously increased and I/M ratios were significantly reduced after the PLM + US treatment. It showed that the hyperplasia VSMC was the acting targets of PTX. Previous documents also showed PTX may inhibit the migration and proliferation of VSMC, even induce the apoptosis of the VSMC^30^,^31^. As expected, the pathologic hyperplasia degree of the damaged artery was reduced effectively, and the area of the lumen was increased significantly by using the PLM + US intervention (Fig. 5A,B). Notably, the efficacy of intervention had no difference between PTX + US group and only US group, indicating that PTX + US did not improve the enough local drug concentration in the artery restenosis region in the absence of MBs. In our study, we also found the lumen area of the iliac artery in the group with only US irradiation was larger than that in the group without any intervention, and the ratios of the intima area and media area were also reduced. In fact, document has demonstrated there were some helpful effects on the vascular structure reconstruction of the damaged artery by low-frequency US irradiation^32^. The possible mechanisms may include US-induced various effects, such as the mechanical effect, US cavitation and the production of the free radicals of ROS^33^,^34^. Furthermore, no obvious toxicity to liver and kidney functions was observed in these animals, indicating PLM + US may provide a safe and effective way to prevent and control the artery restenosis.

## Conclusions

In summary, we developed an US-triggered image-guided drug delivery system for therapeutic applications to iliac restenosis. The resulting PLM had a high drug encapsulation efficiency and US-controllable release capability. More importantly, the PLM could be visually tracked in the iliac artery which not only can favor the identification of iliac restenosis, but also achieve local drug release by low frequency US transducer. Our study provided a promising approach to realize US image-guided PTX delivery for therapeutic applications against iliac restenosis.

## Additional Information

**How to cite this article**: Zhu, X. *et al.* Ultrasound triggered image-guided drug delivery to inhibit vascular reconstruction via paclitaxel-loaded microbubbles. *Sci. Rep.* **6**, 21683; doi: 10.1038/srep21683 (2016).

## Figures and Tables

**Table 1:** The assessment of liver function after PLM + US treatment (n = 10).

Groups	TP (g/L)	ALT (U/L)	AST (U/L)	ALP (U/L)	GGT (U/L)	LDH (U/L)
Normal	50.94 ± 4.02	11.60 ± 5.59	102.40 ± 90.64	116.42 ± 27.47	3.80 ± 2.77	1165.16 ± 764.84
Operation	45.28 ± 4.84	15.40 ± 3.65	76.00 ± 57.68	129.18 ± 28.85	5.00 ± 2.00	419.74 ± 326.97
PLM + US	48.80 ± 5.15	14.60 ± 3.65	91.80 ± 90.53	103.98 ± 27.53	2.40 ± 1.52	769.62 ± 632.86

Note: TP, total protein; ATL, aspartate aminotransferase; AST, aspartate aminotransferase; ALP, alkaline phosphatase; GPT, glutamic pyruvic transaminase; LDH, L-lactate dehydrogenase.

**Table 2:** The assessment of kidney function after PLM + US treatment (n = 10) of the experimental groups

Groups	BUN (mmol/L)	CREA(μmol/L)	URCA(μmol/L)
Normal	18.58 ± 23.88	53.94 ± 30.76	45.02 ± 69.59
Operation	15.63 ± 10.74	63.10 ± 39.77	41.86 ± 36.70
PLM + US	18.76 ± 16.01	48.16 ± 9.61	32.28 ± 26.94

Note: BUN, blood urea nitrogen; CREA, creatinine; URCA, uric acid.

## References

[b1] Poon M., Badimon J. J. & Fuster V. Viewpoint Overcoming restenosis with sirolimus: from alphabet soup to clinical reality. Lancet 359, 619–622 (2002). 11867133 10.1016/s0140-6736(02)07751-6

[b2] Yadav J. *et al.* Protected carotid-artery stenting versus endarterectomy in high-risk patients. New Engl.J. Med. 351, 1493–1501 (2004). 15470212 10.1056/NEJMoa040127

[b3] Costa M. A. & Simon D. I. Molecular basis of restenosis and drug-eluting stents. Circulation 111, 2257–2273 (2005). 15867193 10.1161/01.CIR.0000163587.36485.A7

[b4] Ialenti A. *et al.* Inhibition of in-stent stenosis by oral administration of bindarit in porcine coronary arteries. Arterioscl. Throm.Vas. 31, 2448–2454 (2011). 10.1161/ATVBAHA.111.230078 21852559

[b5] Katsanos K. *et al.* Systematic review of infrapopliteal drug-eluting stents: a meta-analysis of randomized controlled trials. Cardiovasc. Inter. Rad. 36, 645–658 (2013). 10.1007/s00270-013-0578-2 23435741

[b6] Dibra A. *et al.* Paclitaxel-eluting or sirolimus-eluting stents to prevent restenosis in diabetic patients. New Engl. J. Med. 353, 663–670 (2005). 16105990 10.1056/NEJMoa044372

[b7] Albrecht T. *et al.* Reduction of stenosis due to intimal hyperplasia after stent supported angioplasty of peripheral arteries by local administration of paclitaxel in swine. Invest. radiol. 42, 579–585 (2007). 17620941 10.1097/RLI.0b013e31804f5a60

[b8] Scheller B. *et al.* Two year follow-up after treatment of coronary in-stent restenosis with a paclitaxel-coated balloon catheter. Clin. Res. Cardiol. 97, 773–781 (2008). 18536865 10.1007/s00392-008-0682-5

[b9] Tepe G. *et al.* Local taxane with short exposure for reduction of restenosis in distal arteries: THUNDER Trial. N. Engl. J. Med. 358, 689–699 (2008). 18272892 10.1056/NEJMoa0706356

[b10] Werk M. *et al.* Inhibition of restenosis in femoropopliteal arteries paclitaxel-coated versus uncoated balloon: femoral paclitaxel randomized pilot trial. Circulation 118, 1358–1365 (2008). 18779447 10.1161/CIRCULATIONAHA.107.735985

[b11] Doz F. *et al.* Phase I trial and pharmacological study of a 3-hour paclitaxel infusion in children with refractory solid tumours: a SFOP study. Brit. J. cancer 84, 604 (2001). 11237379 10.1054/bjoc.2000.1637 PMC2363793

[b12] Herdeg C. *et al.* Visualization and comparison of drug effects after local paclitaxel delivery with different catheter types. Basic Res. Cardiol. 94, 454–463 (1999). 10651157 10.1007/s003950050161

[b13] Scheller B. *et al.* Paclitaxel balloon coating, a novel method for prevention and therapy of restenosis. Circulation 110, 810–814 (2004). 15302790 10.1161/01.CIR.0000138929.71660.E0

[b14] Speck U. *et al.* Neointima inhibition: comparison of effectiveness of non-stent-based local drug delivery and a drug-eluting stent in porcine coronary arteries. Radiology 240, 411–418 (2006). 16864669 10.1148/radiol.2402051248

[b15] Speck U. *et al.* Inhibition of restenosis in stented porcine coronary arteries: uptake of paclitaxel from angiographic contrast media. Invest. Radiol. 39, 182–186 (2004). 15076010 10.1097/01.rli.0000116125.96544.64

[b16] Kastrati A. *et al.* ISAR-DESIRE Study Investigators. Sirolimus-eluting stent or paclitaxel-eluting stent vs balloon angioplasty for prevention of recurrences in patients with coronary in-stent restenosis: a randomized controlled trial. Jama. 293, 165–171 (2005). 15644543 10.1001/jama.293.2.165

[b17] Fanggiday J. C., Stella P. R., Guyomi S. H. & Doevendans P. A. Safety and efficacy of drug-eluting balloons in percutaneous treatment of bifurcation lesions the DEBIUT (drug-eluting balloon in bifurcaton utrecht) registry. Catheter. Cardio. Inte. 71, 629–635 (2008). 10.1002/ccd.21452 18360855

[b18] Scheller B. *et al.* Paclitaxel balloon coating, a novel method for prevention and therapy of restenosis. Circulation 110, 810–814 (2004). 15302790 10.1161/01.CIR.0000138929.71660.E0

[b19] Kiessling F., Fokong S., Koczera P., Lederle W. & Lammers T. Ultrasound microbubbles for molecular diagnosis, therapy, and theranostics. J. Nucl. Med. 53, 345–348 (2012). 22393225 10.2967/jnumed.111.099754

[b20] Seewald S. *et al.* Lysophosphatidic acid and intracellular signalling in vascular smooth muscle cells. Atherosclerosis 130, 121–131 (1997). 9126656 10.1016/s0021-9150(96)06055-8

[b21] Takeuchi H. *et al.* Potentiation of C-type natriuretic peptide with ultrasound and microbubbles to prevent neointimal formation after vascular injury in rats. Cardiovasc. Res. 58, 231–238 (2003). 12667966 10.1016/s0008-6363(02)00833-7

[b22] Eggenhofer E. *et al.* Mesenchymal stem cells together with mycophenolate mofetil inhibit antigen presenting cell and T cell infiltration into allogeneic heart grafts. Transpl. Immunol. 24, 157–63 (2011). 21194567 10.1016/j.trim.2010.12.002

[b23] Nabel E. G. CDKs and CKIs: molecular targets for tissue remodelling. Nat. Rev. Drug Discov. 1, 587–598 (2002). 12402499 10.1038/nrd869

[b24] Libby P., Schwartz D., Brogi E., Tanaka H. & Clinton S. K. A cascade model for restenosis. A special case of atherosclerosis progression. Circulation 86, III47–52 (1992). 1424051

[b25] Yan F. *et al.* Therapeutic ultrasonic microbubbles carrying paclitaxel and LyP-1 peptide: preparation, characterization and application to ultrasound-assisted chemotherapy in breast cancer cells. Ultrasound Med. Biol. 37(5), 768–79 (2011). 21458148 10.1016/j.ultrasmedbio.2011.02.006

[b26] Yan F. *et al.* Paclitaxel-liposome-microbubble complexes as ultrasound-triggered therapeutic drug delivery carriers. J. Control. Release 166(3), 246–55 (2013). 23306023 10.1016/j.jconrel.2012.12.025

[b27] Tzu-Yin W., Wilson K. E., Machtaler S. & Willmann J. K. Ultrasound and microbubble guided drug delivery: mechanistic understanding and clinical implications. Curr. Pharm. Biotechnol. 14, 743–752 (2013). 24372231 10.2174/1389201014666131226114611 PMC4084724

[b28] Taniyama Y. *et al.* Development of safe and efficient novel nonviral gene transfer using ultrasound: enhancement of transfection efficiency of naked plasmid DNA in skeletal muscle. Gene ther. 9, 372–380 (2002). 11960313 10.1038/sj.gt.3301678

[b29] Iofina E. *et al.* Sirolimus-and paclitaxel-eluting stents in comparison with balloon angioplasty for treatment of in-stent restenosis. Catheter. cardio. Inte. 64, 28–34 (2005). 10.1002/ccd.20212 15619307

[b30] Herdeg C. *et al.* Local paclitaxel delivery for the prevention of restenosis: biological effects and efficacy *in vivo*. J. Am. Coll. Cardiol. (JACC) 35, 1969–1976 (2000). 10841250 10.1016/s0735-1097(00)00614-8

[b31] Axel D. I. *et al.* Paclitaxel inhibits arterial smooth muscle cell proliferation and migration *in vitro* and *in vivo* using local drug delivery. Circulation 96, 636–645 (1997). 9244237 10.1161/01.cir.96.2.636

[b32] Wang Z. G. *et al.* Irradiating effect of low intensive microwave on restenosis of external iliac artery of rabbit after injure. Chin. J. Bases Clin. General Surg. 12, 595–599 (2005).

[b33] Fitzgerald P. J. *et al.* Intravascular sonotherapy decreases neointimal hyperplasia after stent implantation in swine. Circulation 103, 1828–1831 (2001). 11294798 10.1161/01.cir.103.14.1828

[b34] Rosenfeld E. Non-thermal non-cavitational effects of ultrasound. Ultraschall Med. 24, 40–44 (2003). 12599042 10.1055/s-2003-37411

